# Transcriptomics and Network Pharmacology Reveal the Protective Effect of Chaiqin Chengqi Decoction on Obesity-Related Alcohol-Induced Acute Pancreatitis *via* Oxidative Stress and PI3K/Akt Signaling Pathway

**DOI:** 10.3389/fphar.2022.896523

**Published:** 2022-06-08

**Authors:** Xinmin Yang, Linbo Yao, Mei Yuan, Xiaoying Zhang, Monika A. Jakubowska, Pawel E. Ferdek, Lei Dai, Jingyu Yang, Tao Jin, Lihui Deng, Xianghui Fu, Dan Du, Tingting Liu, David N. Criddle, Robert Sutton, Wei Huang, Qing Xia

**Affiliations:** ^1^ Department of Integrated Traditional Chinese and Western Medicine, Sichuan Provincial Pancreatitis Centre and West China-Liverpool Biomedical Research Centre, West China Hospital, Sichuan University, Chengdu, China; ^2^ Malopolska Centre of Biotechnology, Jagiellonian University, Krakow, Poland; ^3^ Department of Cell Biology, Faculty of Biochemistry, Biophysics and Biotechnology, Jagiellonian University, Krakow, Poland; ^4^ State Key Laboratory of Biotherapy and Cancer Center, West China Hospital, Sichuan University and Collaborative Innovation Center of Biotherapy, Chengdu, China; ^5^ Advanced Mass Spectrometry Center, Research Core Facility, Frontiers Science Center for Disease-related Molecular Network, West China Hospital, Sichuan University, Chengdu, China; ^6^ Department of Cellular and Molecular Physiology, Institute of Translational Medicine, University of Liverpool, Liverpool, United Kingdom; ^7^ Liverpool Pancreatitis Research Group, Liverpool University Hospitals NHS Foundation Trust and Institute of Translational Medicine, University of Liverpool, Liverpool, United Kingdom; ^8^ Institutes for Systems Genetics & Immunology, Frontiers Science Center for Disease-related Molecular Network, West China Hospital, Sichuan University, Chengdu, China

**Keywords:** Chaiqin chengqi decoction, obesity-related acute pancreatitis, pharmacology network analysis, transcriptomics, antioxidant protein response, PI3K/Akt pathway

## Abstract

Obesity-related acute pancreatitis (AP) is characterized by increasing prevalence worldwide and worse clinical outcomes compared to AP of other etiologies. Chaiqin chengqi decoction (CQCQD), a Chinese herbal formula, has long been used for the clinical management of AP but its therapeutic actions and the underlying mechanisms have not been fully elucidated. This study has investigated the pharmacological mechanisms of CQCQD in a novel mouse model of obesity-related alcohol-induced AP (OA-AP). The mouse OA-AP model was induced by a high-fat diet for 12 weeks and subsequently two intraperitoneal injections of ethanol, CQCQD was administered 2 h after the first injection of ethanol. The severity of OA-AP was assessed and correlated with changes in transcriptomic profiles and network pharmacology in the pancreatic and adipose tissues, and further docking analysis modeled the interactions between compounds of CQCQD and their key targets. The results showed that CQCQD significantly reduced pancreatic necrosis, alleviated systemic inflammation, and decreased the parameters associated with multi-organ dysfunction. Transcriptomics and network pharmacology analysis, as well as further experimental validation, have shown that CQCQD induced Nrf2/HO-1 antioxidant protein response and decreased Akt phosphorylation in the pancreatic and adipose tissues. *In vitro*, CQCQD protected freshly isolated pancreatic acinar cells from H_2_O_2_-elicited oxidative stress and necrotic cell death. The docking results of AKT1 and the active compounds related to AKT1 in CQCQD showed high binding affinity. In conclusion, CQCQD ameliorates the severity of OA-AP by activating of the antioxidant protein response and down-regulating of the PI3K/Akt signaling pathway in the pancreas and visceral adipose tissue.

## Introduction

Acute pancreatitis (AP) is an inflammatory disease of the pancreas (Mederos et al., 2021), which shows a steady rise in the global incidence over the last 50 years (Iannuzzi et al., 2021). The clinical manifestation of AP varies from asymptomatic/mild to severe cases characterized by extensive pancreatic necrosis, multi-organ failure, and significant mortality (Garg and Singh, 2019). The pathogenesis of AP is complex, and includes several factors, such as premature trypsinogen activation, dysregulated calcium signaling, oxidative/endoplasmic reticulum stress, impaired autophagy, and mitochondrial dysfunction (Gukovskaya et al., 2017; Lee and Papachristou, 2019). Despite the significant prevalence of the disease, currently, there is no internationally-approved pharmacological treatment against AP (Moggia et al., 2017). Therefore, alternative therapeutic strategies to improve the outcomes of AP patients are urgently needed.

Obesity has emerged as an alarming health problem, particularly in developed countries. Overweight bears the risk of cardiometabolic complications, diabetes, cancer, and other diseases (Collaborators et al., 2017; Bluher, 2019), which may cause young-age disability or even death. A growing body of evidence associates obesity with the prevalence (Aune et al., 2021) and severity of AP (Dobszai et al., 2019). The risk factors related to obesity include the formation of gallstones, diabetes, hypertriglyceridemia, and weight loss interventions, all of which may promote lipolysis of the visceral adipose tissue and free fatty acid-mediated lipotoxicity, thus worsening the clinical outcomes of AP (Khatua et al., 2017). Inhibition of pancreatic lipase has been shown to decrease free fatty acid release and the severity of the disease (Navina et al., 2011; de Oliveira et al., 2020). Since visceral fat is implicated in obesity-related AP, new therapeutic strategies centered on the abdominal adipose tissue are an important medical avenue to explore.

Chaiqin chengqi decoction (CQCQD), is a classical traditional Chinese medicine-based herbal formula (Liang et al., 2021). This medicine has been routinely used to treat AP patients for over 40 years in the West China Hospital (Chengdu, Sichuan Province, China), one of the largest pancreatic centers, where more than 2000 AP patients are treated by an integrated approach that combines traditional Chinese and Western medicine (Jin et al., 2020; Li et al., 2020). Treatment with CQCQD can improve the outcomes of AP patients of different etiologies. However, the exact pharmacological mechanism still remains to be elucidated. In a mouse model of AP, our group has recently shown that CQCQD suppresses pancreatic inflammation, reduces acinar cell necrosis and systemic injury *via* modulation of the substance P/neurokinin-1 receptor (Han et al., 2021) and Toll-like four receptor/inflammasome (Wen et al., 2020) signaling pathways.

In the present study, we have investigated the pharmacological mechanisms of CQCQD in a newly established mouse model representing obesity and acute alcohol intake synergistically causes AP (Yang et al., 2022). By integrating the transcriptomic data from the pancreatic and adipose tissues, network pharmacology was constructed to identify the potential protein targets of CQCQD. Subsequently, key molecular mechanisms were validated *in vivo* and *in vitro*. Finally, the interactive activities between CQCQD components and the key target proteins were proposed as a result of docking analysis.

## Materials and Methods

### Ethics and Animals

Male C57BL/6J mice were purchased from Beijing Huafukang Bioscience Co., Ltd (Beijing, China). Animals were maintained at 22 ± 2°C with a 12 h light-dark cycle. Mice received standard laboratory chow, and water was freely accessible throughout the experiment procedures. All animal experiments were approved by the Animal Ethics Committee of the West China Hospital, Sichuan University (20211086A).

### Materials and Reagents

Chow diet (CD; 10 kcal % fat, H10010) and high-fat diet (HFD; 60 kcal % fat, H10060) were from Beijing Huafukang Bioscience Co., Ltd. Ethanol (Sigma-Aldrich) was dissolved in sterile saline at 37.5% concentration (v/v) before use. Substrate for myeloperoxidase (MPO) activity 3,3,5,5-tetramethylbenzidine was from Sigma-Aldrich. Interleukin (IL)-6 enzyme-linked immunosorbent assay was from R&D Systems (Shanghai, China). Propidium iodide (PI), hoechest 33,342, and CM-H2DCFDA were from Thermo Fisher Scientific (Shanghai). Hydrogen peroxide (H_2_O_2_) was from Sinopharm Chemical Reagent Co., Ltd (Shanghai). Anti-nuclear factor erythroid 2-related factor 2 (Nrf2) antibody and anti-heme oxygenase-1 (HO-1) antibody were from Abcam (Shanghai), Akt Rabbit mAb and phosphor-Akt Rabbit mAb were from CST (Shanghai), anti-β-actin antibody was from Proteintech (Shanghai). All other reagents were purchased from Sigma-Aldrich (Shanghai) if not stated otherwise.

### CQCQD Preparation

The raw materia medica of CQCQD was resourced from Sichuan Hospital of Traditional Chinese Medicine (Chengdu, Sichuan, China). CQCQD formula consisted of *Rheum palmatum* L (Da Huang in Chinese, 20 g), Na_2_SO_4_.10H_2_O (Mang Xiao, 20 g), *Magnolia officinalis* Rehd. et Wils (Hou Pu, 15 g), *Citrus aurantium* L (Zhi Shi, 15 g), *Bupleurum marginatum* Wall. ex DC (Chai Hu, 15 g), *Scutellaria baicalensis* Georgi (Huang Qin, 15 g), *Artemisia capillaris* Thunb (Yin Chen, 15 g), and *Gardenia jasminoides Ellis* (Zhi Zi, 20 g). The extraction process and the quality control of CQCQD (D8) has been evaluated by Ultra high-performance liquid chromatography (UHPLC) fingerprinting in our recent publication (Liang et al., 2021) (Supplementary Figures S1A,B).

### AP Model Induction and Treatment

Mice of 4–5 weeks old were randomly assigned into CD and HFD groups, fed with normal chow or high-fat diet for 12 weeks, respectively. Then, ethanol (EtOH) at a dose of 2 g/kg was administered by two intraperitoneal injections 1 h apart, a slight modification of previous ethanol treatment regimens (Huang et al., 2014; Maleth et al., 2015; Wang et al., 2016). The acute EtOH administration in obese mice aimed to establish an obesity-related and alcohol-induced AP (OA-AP) model (Yang et al., 2022), which mimics a clinical scenario, whereby alcoholism increases the risk of developing AP in obese people (Lai et al., 2011). Control lean mice received equal volumes of saline injections. We have previously shown that EtOH alone at this range of doses only caused mild pancreatic edema in lean mice without discernible neutrophil infiltration and acinar cell necrosis (Huang et al., 2014). Therefore, we did not employ this group of control mice for the subsequent experiments.

Regarding the dose of CQCQD, it has been demonstrated in our recently published study that the single clinically-equivalent dose of CQCQD (5.5 g/kg) 3 times is more effective than other doses (Liang et al., 2021; Huang et al., 2022). Therefore, in the treatment group, obese mice were given oral gavage of CQCQD (5.5 g/kg) 3 times every 2 h, starting 2 h after the first EtOH injection. Animals were sacrificed 12 h after the first EtOH/saline injection, and the blood and relevant organs were collected for downstream analyses.

### AP Severity Assessment

AP severity assessment including pancreatic histopathology (see detailed scoring system in Supplementary Figure S2), pancreatic and lung MPO activities, and serum IL-6 was described in detail in our previous studies (Huang et al., 2014; Huang et al., 2017; Wen et al., 2020; Han et al., 2021). Serum biochemical indices including urea, creatinine, alanine aminotransferase (ALT), aspartate aminotransferase (AST), lactate dehydrogenase (LDH), and ionized Ca^2+^ were determined using an automatic biochemical analyzer (Roche Cobas 8,000; Shanghai).

### Transcriptome Analysis

Total RNA was isolated from the pancreas and epididymal adipose tissue using Trizol reagent. RNA degradation and contamination were monitored on 1% agarose gels. RNA purity was checked using the NanoPhotometer^®^ spectrophotometer (IMPLEN, Westlake Village, CA). RNA integrity was assessed using the RNA Nano 6000 Assay Kit of the Bioanalyzer 2,100 system (Agilent Technologies, Santa Clara, CA). Then, sequencing libraries were generated using NEBNext^®^ UltraTM RNA Library Prep Kit for Illumina^®^ (New England Biolabs, Ipswich, MA) following the manufacturer’s recommendations, and index codes were added to attribute sequences to each sample. The clustering of the index-coded samples was performed on a cBot Cluster Generation System using TruSeq PE Cluster Kit v3-cBot-HS (Illumina) according to the manufacturer’s instructions. After cluster generation, the library preparations were sequenced on an Illumina Novaseq platform and 150 bp paired-end reads were generated.

For RNA-seq data analysis, raw data were filtered and adapters were cut using Trim galore software (0.6.6) and quality control was performed using fastp (0.20.1). Then clean reads were mapped to the GRCm38 mouse reference genome using the Star program (2.7.4a). Finally, the featureCounts (2.0.1) software was used to quantify reads. The difference analysis was carried out using the DEseq2 (1.30.0) package in R (4.0.1), and the statistical significance of differentially expressed genes (DEGs) was assessed by the adjusted P cutoff value of 0.05 and fold change cutoff value of 1.5. Gene Ontology (GO) and Kyoto Encyclopedia of Genes and Genomes (KEGG) enrichment analysis of DEGs was conducted using clusterProfiler (3.18.1).

### Immunohistochemistry and Western Blot

Methods for immunohistochemical staining and Western blotting can be found in our previous studies (Wen et al., 2020; Han et al., 2021).

### Network Pharmacology Analysis

A total of 22 Q-markers (synephrine, geniposidic acid, salidroside, coniferin, syringin, geniposide, rutin, narintin, naringin, hesperidin, sennoside A, baicalin, wogonoside, physcion, aloe emodin, bacalein, sinensetin, chrysin, rhein, honokiol, magnolol, and emodin) were identified in our recent study (Liang et al., 2021) and the concentration of each component is presented in the Supplementary Table S1. For each compound, putative targets were acquired from three databases: STITCH (Search Tool for Interactions of Chemicals, http://stitch.embl.de/), ETCM (http://www.tcmip.cn/ETCM/), and STP (SwissTargetPrediction, http://www.swisstargetpred iction.ch). Targets obtained from the three databases were then combined and de-duplicated to obtain the final CQCQD targets. For disease target identification, OA-AP associated targets were obtained based on the transcriptome analysis. Genes related to AP were retrieved and integrated from four databases: GeneCards (https://www.genecards.org/), DisGeNET (https://www.disgenet.org/), OMIM (Online Mendelian Inheritance in Man, https://omim.org/), MalaCards (https://www.malacards.org/).

Network construction and functional enrichment analysis: CQCQD targets were further merged with OA-AP targets, and the overlapping targets identified as CQCQD-regulated OA-AP targets. Cytoscape (3.7.1, https://www.cytoscape.org/) was used to establish the “compound-target” network. In addition, the protein-protein interaction (PPI) network of overlapping targets was constructed by STRING database (https://string-db.org/), and then also visualized with Cytoscape. Functional enrichment analysis of overlapping targets, including GO and KEGG pathway enrichment analysis, was conducted by clusterProfiler (3.18.1) and R (4.0.3). *p* < 0.05 was considered statistically significant.

### Acinar Cell Isolation, Treatment, and Cell Injury Assessment

Mouse pancreatic acinar cells were freshly isolated using a collagenase IV digestion procedure as previously described (Huang et al., 2014; Huang et al., 2017). Cells were pre-treated with CQCQD (5 mg/ml) (Wen et al., 2020) for 30 min, followed by co-incubation with H_2_O_2_ (1 mM) or solvent control for another 30 min with gentle shaking (50 rpm) at room temperature. Reactive oxygen species (ROS; H2-DCFDA, 10 μM) and necrotic cell death (PI, 1 μM) were measured using a plate reader (BMG CLARIOstar; Offenburg, Germany) as previously described (Du et al., 2018; Zhang et al., 2018).

### Molecular Docking Studies

Molecular docking is a theoretical simulation method that models the interactions between compounds and proteins to predict the binding mode and affinity. Molecular docking studies were performed in AutoDock (4.2) and mgltools (1.5.6). The human AKT1 (P31749) protein structure was obtained from the alpha fold protein structure database (https://alphafold.ebi.ac.uk/). All small molecular structures were obtained from the TCMSP (Traditional Chinese Medicine Database and Analysis Platform, https://tcmsp-e.com/) in the corresponding mol2 file. During the pre-docking process, all the polar hydrogen atoms were added, and the water molecules were removed. Each molecule was docked with the protein for 50 times, and the lowest binding energy was selected to display the result at least once. The docking score was expressed as the binding energy. Lower binding energy reflects stronger interaction between the compound and protein. Finally, PyMOL software (2.4.1) was used to visualize the docking results. The python environment on which the above software depends is Python (3.7.4).

### Statistical Analysis

All data are presented as means ± SEM. Statistical analysis was carried out using GraphPad Prism 8.4.1. For two-group comparisons, mean differences were analyzed by a two-tailed Student’s *t-*test. For multi-group comparisons, mean differences were analyzed by one-way ANOVA with a Tukey’s multiple comparison post-hoc test. *p* values <0.05 were considered significant.

## Results

### CQCQD Reduces Pancreatitis Indices in OA-AP and Multi-Organ Dysfunction

**FIGURE 1:**
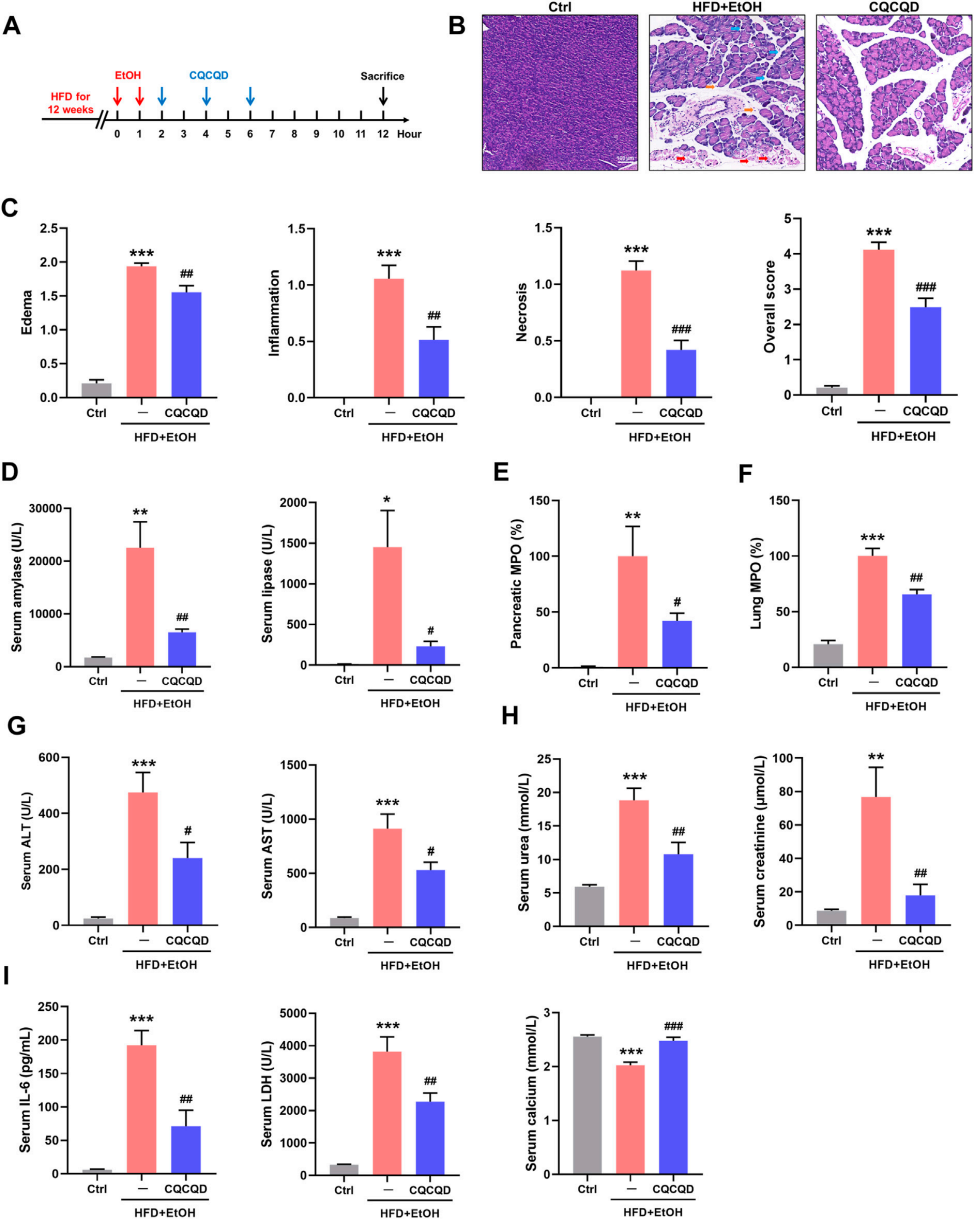
CQCQD ameliorates the severity of OA-AP in a mouse model. **(A)** Experimental protocol of this study. **(B)** Representative H&E images of pancreatic sections (blue arrows indicate edema, orange arrows indicate inflammation, and red arrows indicate necrosis). **(C)** Pancreatic histopathology scores (edema, inflammatory infiltration, acinar cell necrosis, and the overall score). **(D)** Levels of serum amylase and lipase. **(E)** Pancreatic myeloperoxidase (MPO). **(F)** Lung MPO. **(G)** Serum alanine aminotransferase (ALT) and aspartate aminotransferase (AST). **(H)** Serum urea and creatinine levels. **(I)** Serum interleukin-6 (IL-6), lactate dehydrogenase (LDH), and ionized calcium. All data are presented as means ± SEM of 6–10 animals per group. Ctrl vs. OA-AP: *p < 0.05, **p < 0.01, ***p < 0.001; OA-AP vs. OA-AP + CQCQD: ^#^ *p* < 0.05, ^##^ *p* < 0.01, ^###^ *p* < 0.001.

The experimental pipeline of the OA-AP model and administration of CQCQD is shown in Figure 1A. Compared to the control mice, mice from the OA-AP group developed features of pancreatic injury (separated acinar lobules, intra-parenchymal neutrophil infiltration, and patchy acinar cell necrosis; Figure 1B), which was reflected by markedly increased corresponding histopathological scores (Figure 1C), the elevation of serum amylase and lipase levels (Figure 1D) as well as pancreatic MPO (Figure 1E). The OA-AP model was also associated with dramatically raised parameters of multiple organ dysfunction in the lung (Figure 1F), liver (Figure 1G), and kidney (Figure 1H); as well as deranged general severity indices (Figure 1I) including IL-6, LDH, and ionized calcium. Administration of CQCQD significantly ameliorated pancreatic injury (Figures 1B–E) and multi-organ dysfunction (Figures 1F–H) as well as normalized general severity indices (Figure 1I).

These data collectively show that CQCQD was protective against OA-AP, manifested as a reduction in pancreatic necrosis and protection against multi-organ dysfunction.

### Transcriptome Analysis Reveals the Signaling Pathways Involved in the OA-AP Pathogenesis

**FIGURE 2:**
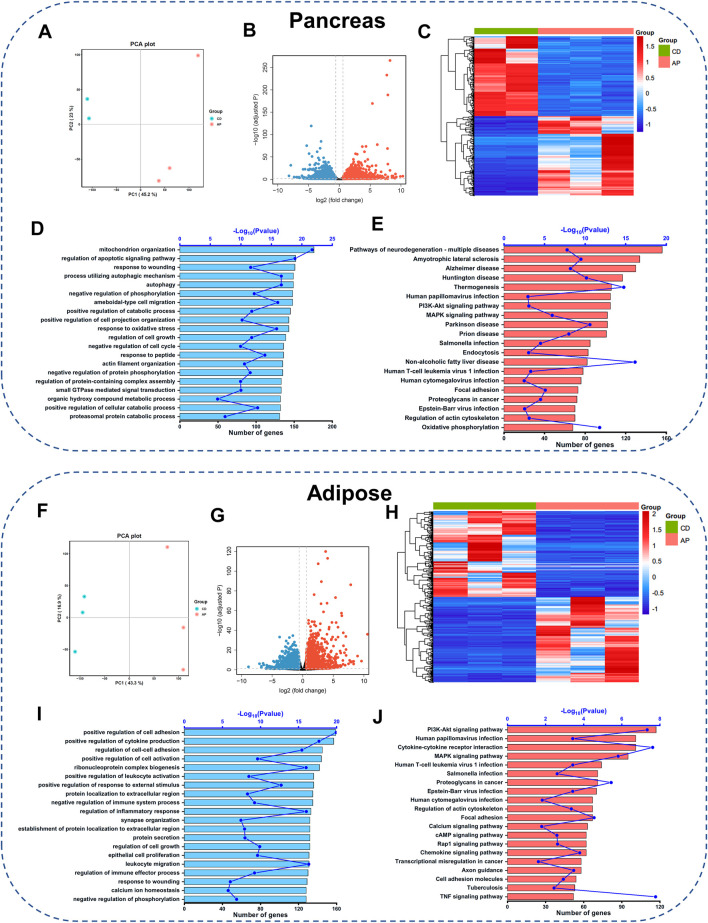
Gene expression profiling in the pancreatic and adipose tissues of OA-AP mice. Transcriptome analysis of differentially expressed genes (DEGs) between OA-AP group and control group in the pancreatic tissue **(A–E)** and adipose tissue **(F–J)**, respectively. **(A,F)** Principal component analysis. **(B,G)** Volcano Plot. **(C,H)** Heatmap. **(D,I)** GO analysis shows the top 20 terms based on enrich factor arrangement. **(E,J)** KEGG pathway enrichment analysis shows the top 20 terms based on enrichment factor arrangement.

We next carried out the transcriptome analysis in the pancreatic tissue to understand the molecular mechanisms underlying pancreatic injury during OA-AP progression. Compared to the control group (lean mice that received only saline injections), OA-AP induced different expressions of a total of 4,171 genes (DEGs) in the pancreas; of these DEGs, 2,114 were upregulated and 2057 were downregulated (Figures 2A,B and Supplementary Table S2). Further, unsupervised hierarchical clustering analysis of the top 500 DEGs showed a substantial difference between the OA-AP and the control pancreatic tissues (Figure 2C). Subsequently, GO enrichment analysis indicated the changes in biological processes related to mitochondrial organization, regulation of the apoptotic signaling pathway, processes utilizing autophagic mechanisms, autophagy, negative regulation of phosphorylation (all *p* < 0.05; Figure 2D and Supplementary Figures S3A,B). KEGG analysis revealed that neurodegeneration, the phosphatidylinositol 3-kinase-Akt (PI3K/Akt) signaling pathway, mitogen-activated protein kinase (MAPK) signaling pathway, focal adhesion, and oxidative phosphorylation were the top-ranked signaling pathways (all *p* < 0.05, Figure 2E and Supplementary Figures S3C,D).

Since the adipose tissue plays a crucial role in obesity-related AP, we included the adipose tissue transcriptomics to further clarify the potential pathological mechanisms of OA-AP. Volcano plot (Figures 2F,G and Supplementary Table S3) and heatmap (Figure 2H) analyses showed substantial differentiation between OA-AP and control visceral adipose tissues. GO enrichment analysis indicated the changes in the biological processes such as the positive regulation of cell adhesion, positive regulation of cytokine production, and others (Figure 2I and Supplementary Figures S3E,F). KEGG pathway analysis highlighted the involvement of the PI3K-Akt signaling pathway, cytokine-cytokine receptor interaction, MAPK signaling pathway, and focal adhesion (Figure 2J and Supplementary Figures S3G,H).

### Pharmacology Network Construction Between CQCQD Targets and DEGs of OA-AP

**FIGURE 3:**
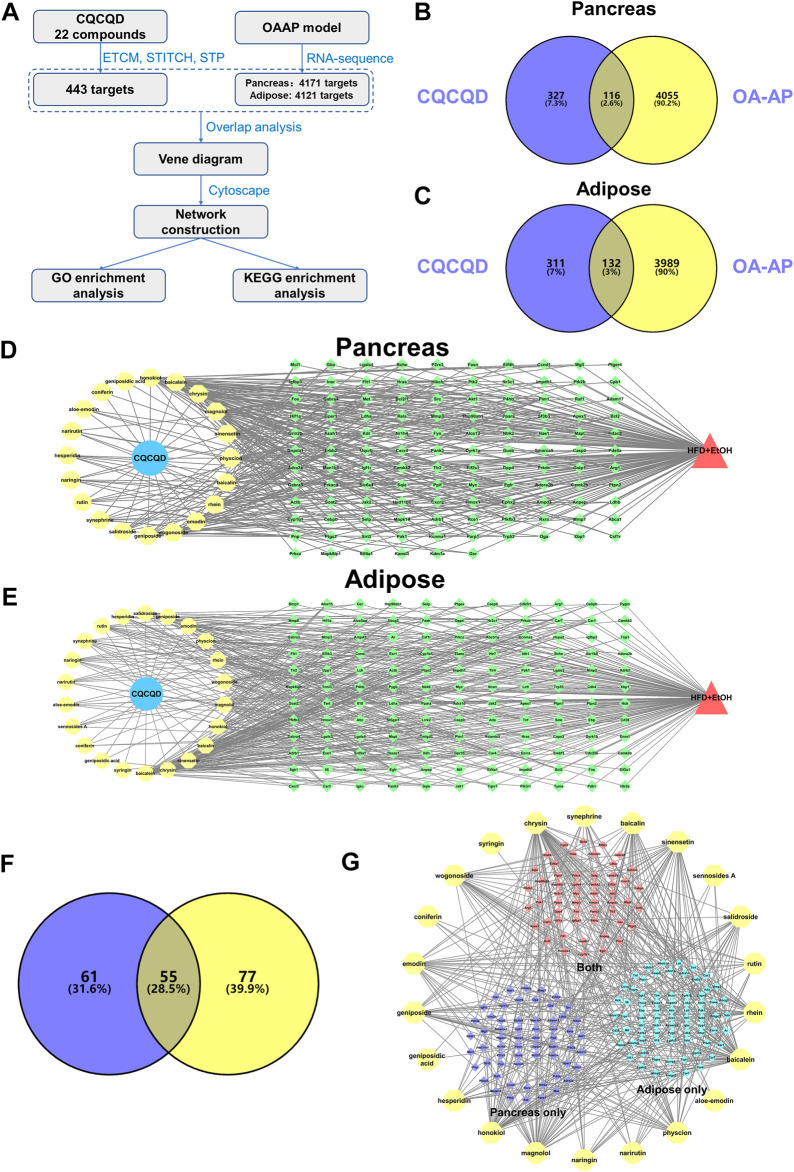
Network targets of CQCQD on OA-AP both in the pancreatic and adipose tissues. **(A)** The protocol of network pharmacology analysis in this study. **(B,C)** Venn diagram shows overlapped targets between CQCQD and OA-AP in the pancreatic tissue **(B)** and adipose tissue **(C)**. **(D,E)** The compound-target network was established in the pancreatic tissue **(D)** and adipose tissue **(E)**. **(F)** Venn diagram shows overlapped CQCQD-regulated targets between the pancreatic tissue and adipose tissue. **(G)** The compound-target network is based on the pancreatic tissue and adipose tissue.

In our study, we employed online databases that identified a total of 443 targets of 22 compounds from CQCQD (Supplementary Table S4), while the targets related to the disease were obtained from the transcriptomic analysis of the OA-AP model. The network pharmacology analysis was performed according to a pre-defined protocol (Figure 3A). Firstly, the Venn diagram showed overlapped targets between CQCQD and OA-AP. Based on these, a compound-target network was established, and related functional enrichment analysis was carried out. As a result, 116 and 132 overlapped targets were selected as potential therapeutic targets of CQCQD in the pancreatic (Figure 3B) and adipose tissues, respectively (Figure 3C). After discarding the compounds with no predicted target available, 20 compounds with 116 predicted targets were obtained in the pancreas (Figure 3D), and 22 compounds with 132 predicted targets in the adipose tissue (Figure 3E). In addition, we overlapped the targets of CQCQD from the pancreatic and adipose tissue (Figure 3F). There were 55 common targets, with the other 61 and 77 targets specific for the pancreas or adipose tissue, respectively. The network “compounds-targets-tissue” is shown in Figure 3G.

### GO and KEGG Pathway Enrichment Analysis of CQCQD-Regulated Targets

**FIGURE 4:**
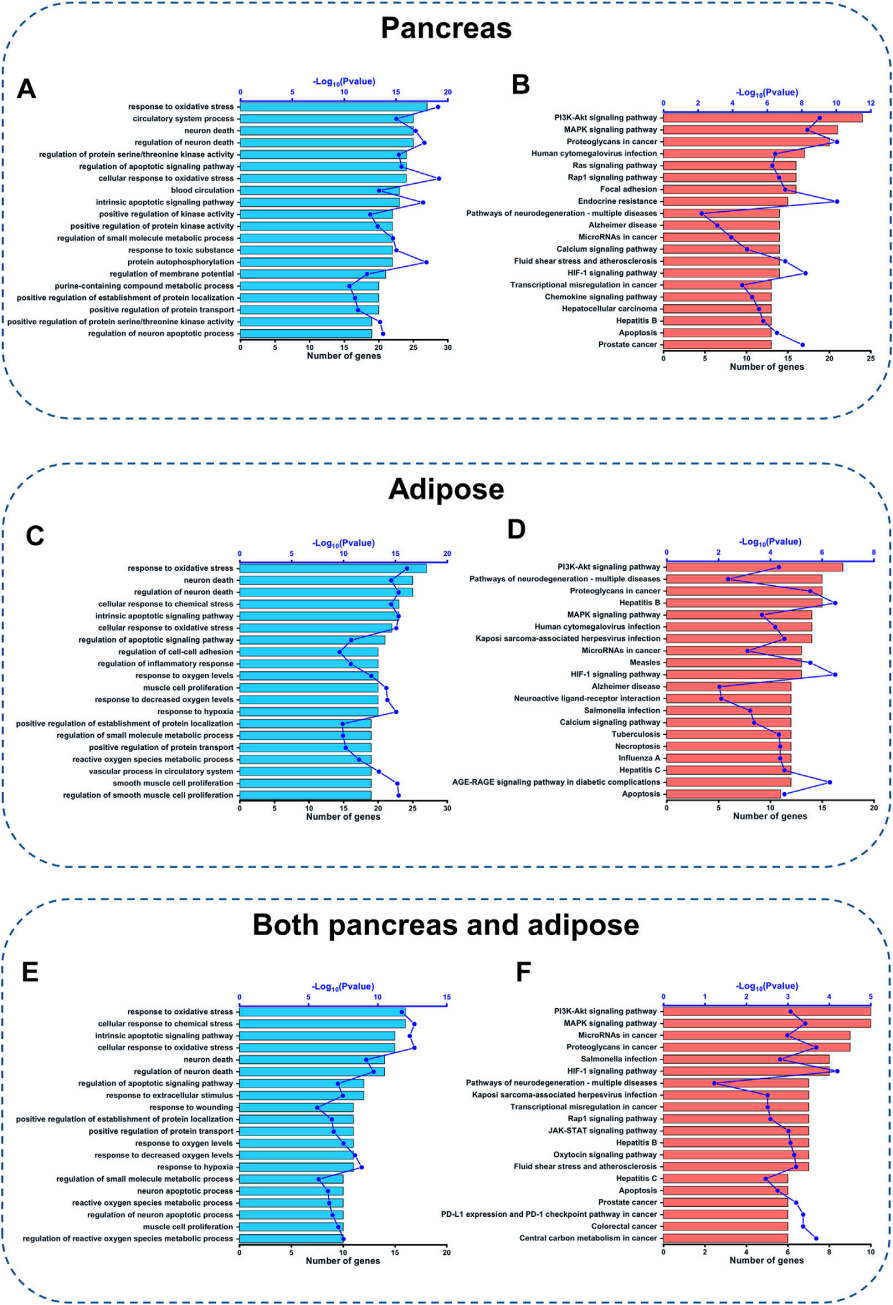
GO function and KEGG pathway analysis of CQCQD-regulated targets. **(A,C,E)** Top 20 biological processes returned by the GO analysis of the pancreatic targets **(A)**, adipose tissue targets **(C)**, and common targets of the pancreatic tissue and adipose tissue **(E)**. **(B,D,F)** Top 20 KEGG pathways of the pancreatic targets **(B)**, and adipose tissue targets **(D)**, and common targets of the pancreatic tissue and adipose tissue **(F)**.

To further investigate the detailed functions of the above CQCQD-regulated targets in the pancreas and adipose tissues, GO function and KEGG pathway enrichment analyses were carried out. For the targets specific for the pancreas, GO enrichment analysis mainly returned genes related to the response to oxidative stress, neuronal death, regulation of apoptotic signaling pathway, and other processes (Figure 4A). KEGG enrichment analysis showed that the pancreatic targets were closely related to the PI3K/Akt, MAPK, and Ras signaling pathways, hypoxia-inducible factor 1 signaling pathway, and apoptosis (Figure 4B). Concomitantly, GO enrichment analysis showed that the targets specific for the adipose tissue are potentially engaged in the response to oxidative stress, neuronal death, and cellular response to chemical stress (Figure 4C); whereas KEGG enrichment analysis highlighted the involvement of the PI3K/Akt and MAPK signaling pathways, calcium signaling and others (Figure 4D). Finally, GO enrichment analysis of the common targets of the pancreas and adipose tissue showed that these genes are engaged in the response to oxidative stress, cellular response to chemical stress, and intrinsic apoptotic signaling pathway (Figure 4E). KEGG enrichment analysis of those targets pointed towards pathways such as PI3K/Akt, MAPK, and Rap1 (Figure 4F). Interestingly, we also found that the response to oxidative stress and the PI3K/Akt signaling pathway were among the top20 ranked pathways returned by the GO and KEGG analyses of the targets common for both OA-AP and AP (Supplementary Table S5 and Supplementary Figures S4A–C). Collectively, these results suggest that the protective effects of CQCQD against OA-AP were likely mediated by induction of antioxidant protein response and via reduced PI3K-Akt signaling.

### CQCQD Decreases Oxidative Stress and Downregulates PI3K-Akt Signaling in Experimental OA-AP

**FIGURE 5:**
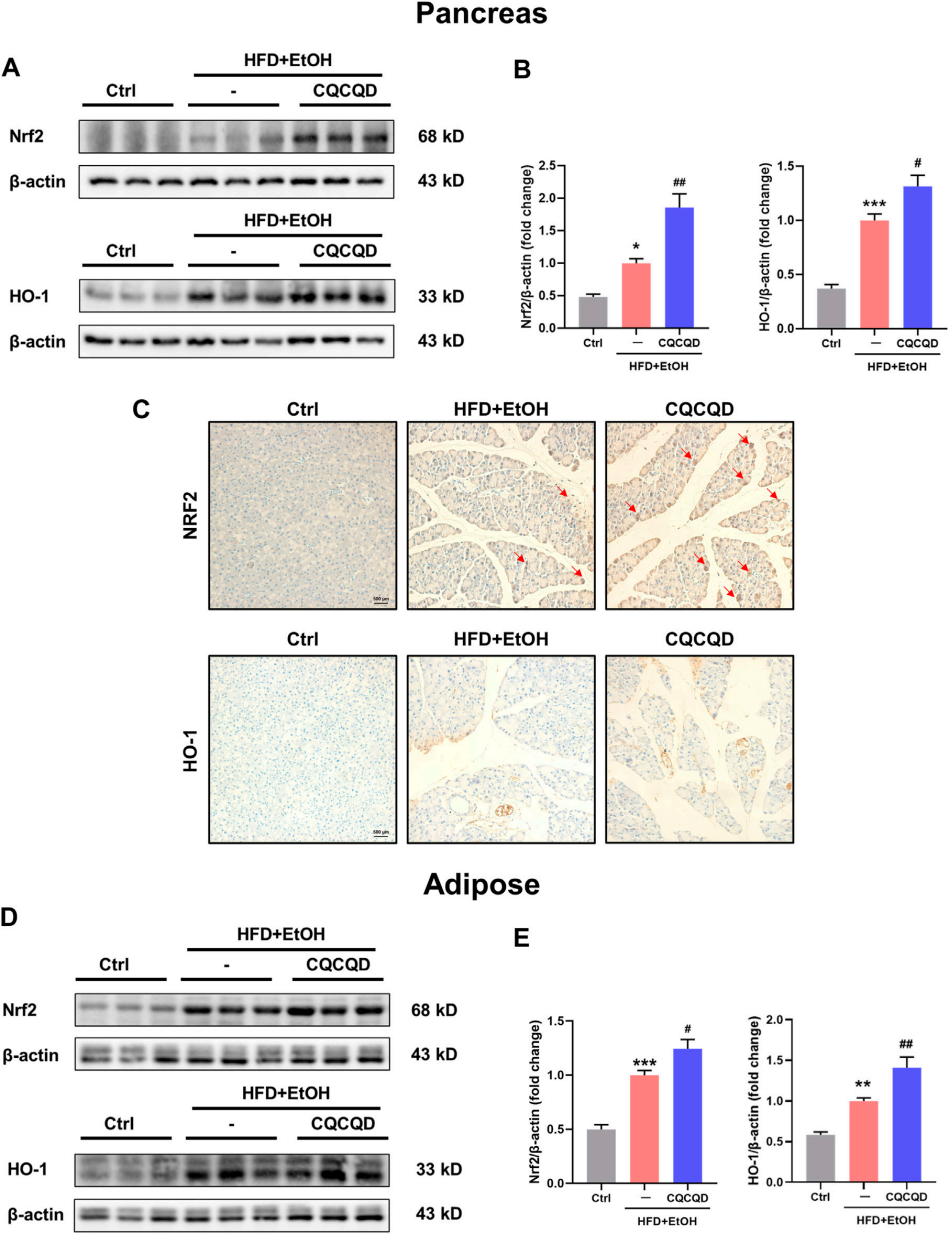
CQCQD enhances the antioxidant protein response in the pancreas and adipose tissue from OA-AP mice. **(A,D)** Representative Western-blot images for Nrf2 and HO-1 in the pancreatic tissues **(A)** and adipose tissues **(D)**. **(B,E)** Quantification of Western-blot images for Nrf2 and HO-1 for the pancreatic tissues **(B)** and adipose tissues **(E)**. **(C)** Representative immunohistochemical staining for Nrf2 and HO-1 in the pancreatic tissue. All data are presented as means ± SEM of six samples per group. Ctrl vs. OA-AP: *p < 0.05, **p < 0.01, ***p < 0.001; OA-AP vs. OA-AP + CQCQD: ^#^ *p* < 0.05, ^##^ *p* < 0.01, ^###^ *p* < 0.001.

To validate the findings from transcriptomics and pharmacology network analyses, we tested the expression of selected proteins involved in oxidative stress and the PI3K-Akt signaling pathway. Two indicators of oxidative stress were chosen: 1) an endogenous key anti-oxidative stress factor Nrf2, a master regulator of the antioxidant protein response; and 2) HO-1, an Nrf2-regulated detoxifying enzyme (Shapiro et al., 2007). Western blot images (Figure 5A) and the corresponding densitometric analysis (Figure 5B) demonstrated that the expression levels of pancreatic Nrf2 and HO-1 proteins were significantly up-regulated in the OA-AP mice compared to control. Following the CQCQD treatment, the expression levels of these proteins were further increased, indicating an enhanced anti-oxidant capacity against free radicals. This was further confirmed by the immunohistochemical staining for Nrf2 and HO-1 (Figure 5C). The control pancreata showed only very weak cytosolic staining patterns of Nrf2 and HO-1, essentially with no signal in the nuclei. In the OA-AP samples, both these proteins were expressed at a much higher level and were characterized by cytosolic and nuclear patterns of localization. The expression was even further elevated in the OA-AP groups treated with CQCQD (Figure 5C). The same patterns of Nrf2 and HO-1 expression were also observed in the visceral adipose tissues (Figures 5D,E).

**FIGURE 6:**
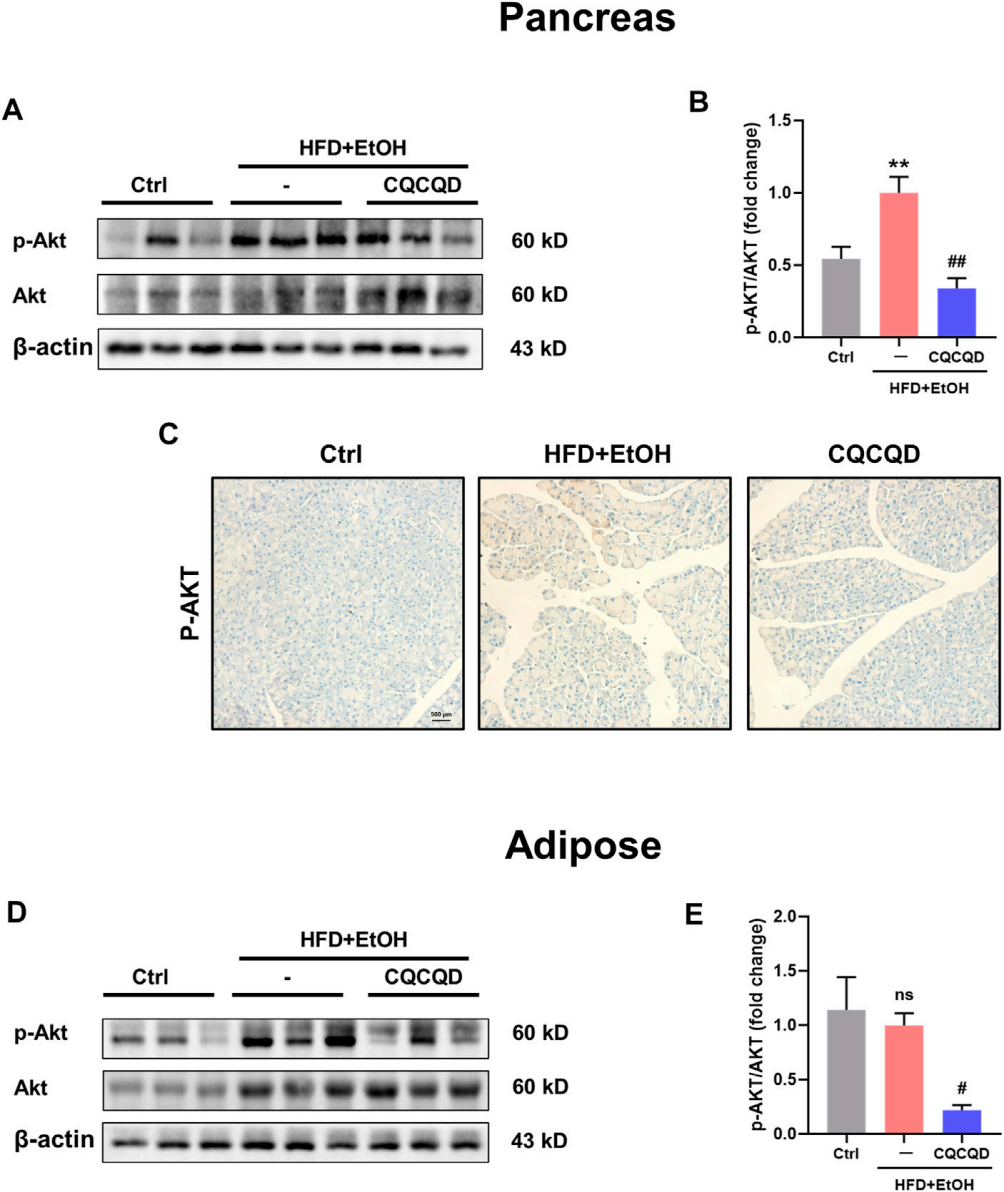
CQCQD decreases Akt phosphorylation in the pancreas and adipose tissue from OA-AP mice. **(A,D)** Representative Western-blot images for p-Akt and total Akt in the pancreatic tissues **(A)** and adipose tissues **(D)**. **(B,E)** Quantification of Western-blot images for p-Akt/Akt of the pancreatic tissues **(B)** and adipose tissues **(E)**. **(C)** Representative immunohistochemical staining for p-Akt in the pancreatic tissue. All data are presented as means ± SEM of six samples per group. Ctrl vs. OA-AP: **p < 0.01; OA-AP vs. OA-AP + CQCQD: ^#^ *p* < 0.05, ^##^ *p* < 0.01.

To verify the involvement of the PI3K/Akt signaling pathway, two protein targets, Akt and phosphorylated-Akt (p-Akt), were selected and presented as the p-Akt/Akt ratio (Chen et al., 2020; Sarker and Steiger, 2020). While the p-Akt/Akt ratio was significantly increased in the OA-AP pancreata, CQCQD markedly decreased Akt phosphorylation to the levels in the OA-AP (Figures 6A,B). This was also evidenced by the immunohistochemical staining for p-Akt in the pancreatic tissue (Figure 6C). While Akt phosphorylation was not affected by OA-AP in the adipose tissue, CQCQD decreased the p-Akt/Akt ratio by approximately 75% compared to OA-AP without treatment (Figures 6D,E).

Both oxidative stress and the PI3K-Akt signaling pathway are associated with programmed death such as apoptosis (Cao et al., 2019). We found that apoptosis was involved in the top20 ranked pathways returned by the GO and KEGG analyses of the CQCQD-regulated targets in the pancreas and adipose tissues (Figures 4A–F). The expression of B-cell lymphoma-2 (Bcl2) and Bcl2-associated X protein of pancreatic tissues were detected to illustrate the effect of CQCQD on the acinar cells. While the Bax/Bcl2 ratio was significantly increased in the OA-AP pancreata, CQCQD markedly decreased this ratio in the OA-AP (Supplementary Figures S5A,B). The pancreatic TUNEL statining also supported CQCQD improved apoptosis in acinar cells (Supplementary Figure S5C).

These data collectively suggest that CQCQD reduced the severity of OA-AP by additional stimulation of the already activated antioxidant protein response (i.e., Nrf2/HO-1 pathways) and at the same time suppressed the PI3K/Akt signaling pathway in the pancreatic and adipose tissues.

### CQCQD Reduces Pancreatic Acinar Cell Death Elicited by Oxidative Stress

**FIGURE 7:**
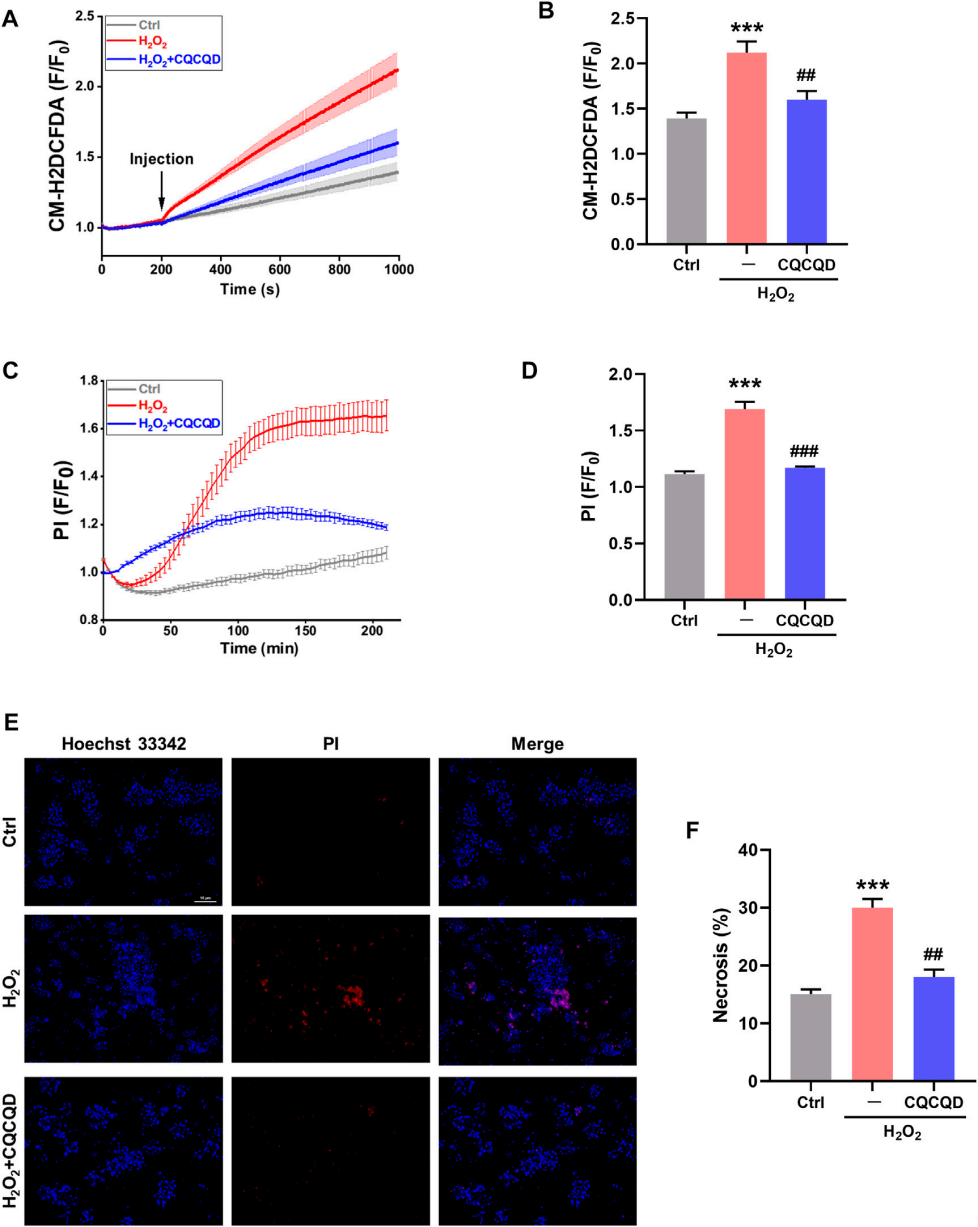
CQCQD decreases ROS production and cell necrosis induced by H_2_O_2_ in mouse pancreatic acinar cells. **(A)** Average traces reflect intracellular ROS signals. **(B)** Summary quantification of ROS production (F/F_0_). **(C)** Typical traces of cell necrosis. **(D)** Summary quantification of cell necrosis (F/F_0_). **(E)** Representative images of pancreatic acinar cells stained with Hoechst 33,342 (blue) and PI (red). **(F)** Quantification of cell necrosis (%). All data are presented as means ± SEM of ≥ 3 repeats per group. Ctrl vs. OA-AP: ***p < 0.001; OA-AP vs. OA-AP + CQCQD: ^##^ *p* < 0.01, ^###^ *p* < 0.001.

The effects of CQCQD were also tested *in vitro* in mouse pancreatic acinar cells. Acinar cells were isolated from lean mice and were pre-incubated with or without CQCQD, followed by treatment with H_2_O_2_ to induce oxidative stress (Huang et al., 2015). While H_2_O_2_ stimulation induced a steady increase in the reactive oxygen species (ROS) generation (Figures 7A,B) and triggered necrotic cell death of acinar cells (Figures 7C,D), CQCQD pre-incubation significantly reduced the recorded ROS signals as well as acinar cell necrosis (Figures 7A–D). The representative images of the PI staining of acinar cells are shown in Figures 7E,F. These results suggest that the protective effects of CQCQD against necrotic cell death in acinar cells are likely mediated by increased detoxification or scavenging of intracellular free radicals (Armstrong et al., 2018).

### Docking Analysis Between CQCQD Compounds and AKT1

**FIGURE 8:**
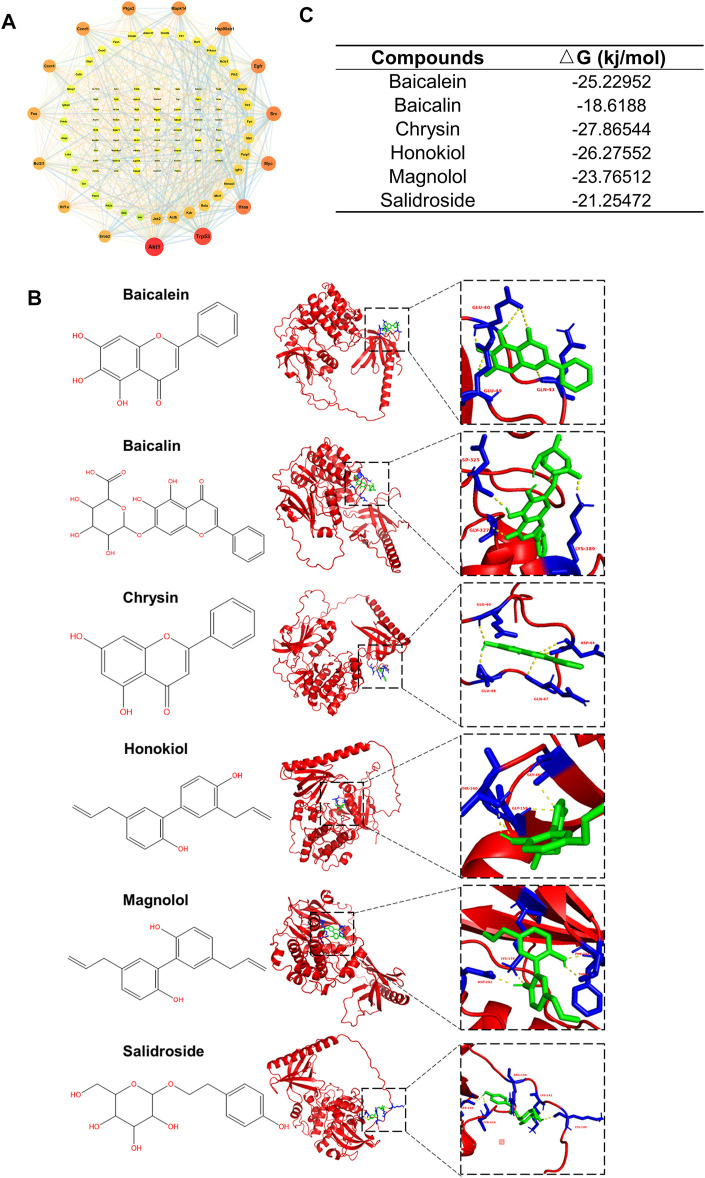
Identification of interactions between CQCQD active compounds and the targets they regulate. **(A)** Interaction network of CQCQD-regulated targets in the pancreatic tissue. **(B)** Modeling interactions of CQCQD compounds and Akt1 by molecular docking. **(C)** The binding affinity between CQCQD compounds and Akt1.

Finally, we narrowed down and explored the active compounds of CQCQD. A PPI network of 116 pancreatic targets of CQCQD was constructed by STRING database, which returned AKT1 as a target of the highest degree value (Figure 8A). Importantly, AKT1 was also one of the key targets common for OA-AP and AP (Supplementary Figure S6). Subsequently, a molecular docking approach was used to predict the interactive activities between CQCQD compounds and AKT1. Based on the network pharmacology, six compounds of CQCQD, i.e., baicalein, baicalin, chrysin, honokiol, magnolol, and salidroside, were found capable of high-affinity binding to AKT1 (Figures 8B,C).

## Discussion

This study has been inspired by promising clinical outcomes in AP patients treated with CQCQD in the West China Hospital. Since obesity is an important risk factor of AP and approximately 21% of all AP patients admitted to our center suffers from obesity-related AP (Jin et al., 2020; Li et al., 2020; Shi et al., 2020), we sought to shed some light on the pharmacological mechanisms underlying the clinical efficacy of CQCQD. For this purpose, we applied a new clinically-relevant mouse model of OA-AP along with transcriptome analysis of the pancreatic and adipose tissues to identify potential gene targets and signaling pathways modulated by active compounds of CQCQD. Further, network pharmacology and molecular biology revealed that the protective effects of CQCQD on OA-AP are likely principally mediated by the antioxidant protein response and inhibition of the PI3K/Akt signaling pathway in both tissues. To the best of our knowledge, this is the first study that has linked the clinical effects of this traditional herbal formula with signaling in adipose tissue. Docking analysis further identified that CQCQD active compounds were capable of binding to Akt1, the protein kinase revealed by our *in silico* analyses.

Recently, obesity has been recognized as an important factor involved in the pathogenesis and progression of AP (Khatua et al., 2017). Alcohol consumption is another important risk factor for AP, although the pathology of alcoholic pancreatitis is still unclear (Alsamarrai et al., 2014; Samokhvalov et al., 2015). A recent epidemiological report (Lai et al., 2011) has shown an increased risk of AP in obesity-related metabolic conditions, such as type 2 diabetes combined with excessive alcohol consumption. Furthermore, previous preclinical studies have demonstrated that alcohol simultaneously supplied with free fatty acids can effectively induce AP in mice, rats, and hamsters (Huang et al., 2014; Maleth et al., 2015; Wang et al., 2016). The above has prompted us to apply a novel AP model induced by obesity and acute alcohol adminstration. Briefly, acute alcohol administration in obese mice caused pancreatic necrosis, systemic inflammation, and multi-organ dysfunction, while there were no significant changes in the histopathology of the pancreata from obese mice injected with saline alone or in lean mice injected with ethanol alone (Yang et al., 2022). This model may therefore reflect the pathophysiology underlying AP in obese patients, who are particularly vunlernable to progress to severe AP and poor clinical outcomes. However, OA-AP model is only suitable for obese mice, which may limit its use. And intraperitoneal injection of EtOH is still different from that of human drinking. Cerulein, a cholecystokinin analog, is the most widely used compound to induce AP (CER-AP) in rodents, which is characterized by overt pancreatic tissue edema, inflammatory cell infiltration, and a ceterin amount of acinar cell necrosis (Yang et al., 2020). Cerulein is often combined with lipopolysaccharide to increase the severity of CER-AP by exaggerating the inflammatory response and multi-organ dysfunction, mimicking AP-associated sepsis (Yang et al., 2020). Different from CER-AP, the OA-AP has much more increased indices for multi-organ dysfunction and the most significant pancreatic pathological change is a preferential acinar cell necrosis (Yang et al., 2022).

We found that in our OA-AP model, CQCQD administered at 5.5 g/kg, an equivalent dose to that regularly used in the clinic (Liang et al., 2021), decreased the pancreatic pathology score, local and systemic inflammation, and other organ injuries. Although previous reports have shown that CQCQD was effective in other preclinical models of AP, this study is the first to provide evidence on CQCQD efficacy in obesity-related pancreatic inflammation of alcoholic etiology.

In this study, we have applied transcriptomics and *in silico* analyses to identify DEGs in the pancreatic and adipose tissues from control and OA-AP mice. Modern methods of network pharmacology have been used to characterize active compounds of the traditional Chinese herbal formula. Predicted targets of 22 active compounds from CQCQD were overlapped with target genes differentially expressed in OA-AP and the “compound-target” network has been constructed. As a result, the antioxidant protein response and the PI3K/Akt signaling pathway have been identified as the key players in the CQCQD-mediated improvement of OA-AP outcomes. The same results were returned by the functional enrichment analysis of transcriptomics and network pharmacology both in the pancreas and adipose tissue. This is particularly important, since previous studies have demonstrated that oxidative stress and the PI3K/Akt signaling may underlie the pathogenesis of AP (Leung and Chan, 2009; Sarker and Steiger, 2020) and other acute inflammatory diseases (Manning and Toker, 2017).

Given that oxidative stress is implicated in AP (Tsai et al., 1998; Leung and Chan, 2009; Booth et al., 2011), antioxidant therapy can be a potential direction to explore further in AP treatment (Esrefoglu, 2012; Armstrong et al., 2013; Jiang et al., 2020). Nrf2/HO-1 pathway is the master regulator of cellular antioxidant responses (Kensler et al., 2007; Ma, 2013). Under stress conditions, Nrf2 translocates into the nucleus and activates antioxidant response elements that regulate gene expression of several enzymes including HO-1 (Kensler et al., 2007; Ma, 2013). Recently, natural or synthetic compounds capable of activating the Nrf2/HO-1 pathway in the pancreas have been reported to alleviate the severity of AP (Dong et al., 2016; Du et al., 2018; Liu et al., 2018). Similarly to cerulein-induced AP (Dong et al., 2016; Du et al., 2018; Liu et al., 2018), in our OA-AP model there was an up-regulation of Nrf2/HO-1, likely resulting from increased oxidative stress during the ongoing tissue inflammation. However, transcriptional activation of the Nrf2/HO-1 pathway by CQCQD, both in the pancreatic and adipose tissues, might be important for efficient detoxification of free radicals and thus could alleviate the progression of AP. In order to verify our findings experimentally, we first tested whether CQCQD could protect against oxidative stress in pancreatic acinar cells, in which premature activation of digestive enzymes causes autodigestion of the organ. CQCQD reduced the H_2_O_2_-elicited ROS signals and cell necrosis in acinar cells, thus confirming its protective anti-oxidant capacity in the cellular mediators of the sterile pancreatic inflammation. Although no clear benefit was found in the clinical trials of classical antioxidants in AP (Armstrong et al., 2013), our study provides the possibility of developing natural alternatives from traditional Chinese medicine.

The important role of the PI3K/Akt signaling pathway in the pathogenesis of inflammatory diseases, including AP, is well established (Lupia et al., 2014; Sarker and Steiger, 2020). Several previous studies implicated the PI3K/Akt pathway in trypsinogen activation, calcium overload, the nuclear translocation of NF-κB and cell apoptosis in the early course of AP, and strategies aim at PI3K inactivation to ameliorate the outcome of AP (Singh et al., 2001; Gukovsky et al., 2004; Abliz et al., 2015). On the other hand, Malagola et al. found that pharmacologic, or genetic inhibition of the Akt1 decreased acinar proliferation and exacerbated acinar-to-ductal metaplasia formation in the late course of AP following inflammatory insults (Chen et al., 2020). The PI3K/Akt pathway also plays an important role in insulin signaling cascade of adipose tissue by promoting glucose utilization, protein synthesis, and lipogenesis as well as inhibiting lipolysis (Huang et al., 2018). Furthermore, PI3K/Akt pathway also modulated Nrf2 signaling by regulating Nrf2 phosphorylation, activity, and degradation (Yu and Xiao, 2021). In this study, we show that CQCQD markedly decreased Akt phosphorylation and thus inhibited the PI3K/Akt signaling our OA-AP model. Out of the active compounds of CQCQD, baicalein, baicalin, chrysin, honokiol, magnolol, and salidroside, all demonstrated marked potential for high affinity binding to AKT1, with chrysin being the most potent. While previous studies have reported that some of these compounds can improve AP in the *in vivo* or *in vitro* models (Li et al., 2018; Pu et al., 2019), the evidence that CQCQD components modulate oxidative stress or the PI3K/Akt pathway is marginal. In different diseases, honokiol was used as an inhibitor of Akt activation (Zhai et al., 2005), and chrysin was postulated to modulate the PI3K/Akt pathway (Zhou et al., 2021). In our study, out of a large pool of possible biological targets tested, it was the PI3K/Akt pathway that has emerged as the major mechanism regulated in OA-AP by the CQCQD active compounds.

## Conclusion

This study is the first to explore how CQCQD herbal medicine, used in the pancreas clinics, ameliorates obesity-related and alcohol-induced AP via modulation of the antioxidant protein response and the PI3K/Akt pathway, in both the pancreatic and adipose tissues. Our findings provide a solid foundation for the applications of CQCQD or its modified formulas not only in traditional Chinese medicine but also in general clinical practice.

## Data Availability

The datasets presented in this study can be found in online repositories. The names of the repository/repositories and accession number(s) can be found below: 220408 PM: https://www.ncbi.nlm.nih.gov; GSE200061.

## Supplementary Material

Supplementary Figure S1 Fingerprints and chemometric analysis of content in 22 Q-markers for CQCQD. **(A)** Ultra high-performance liquid chromatography(UHPLC) fingerprints for 10 batches (D1∼D10) of CQCQD, and batch R is the simulated chromatogram (PMID: 33740732). **(B)** the representative total ion chromatograms (TIC) of CQCQD (D8, used in this study).

Supplementary Figure S2 Pancreatic histopathology scoring system.

Supplementary Figure S3 GO function and KEGG pathway analysis of the up-regulated genes and the down-regulated genes in the pancreatic and adipose tissues of OA-AP. Top 20 biological processes returned by the GO analysis of the up-regulated genes **(A)** and the down-regulated genes **(B)** in the pancreatic tissue. Top 20 KEGG pathways of the up-regulated genes **(C)** and the down-regulated genes **(D)** in the pancreatic tissue. Top 20 biological processes returned by the GO analysis of the up-regulated genes **(E)** and the down-regulated genes **(F)** in the adipose tissue. Top 20 KEGG pathways of the up-regulated genes **(G)** and the down-regulated genes **(H)** in the adipose tissue.

Supplementary Figure S4 Comparison of the gene targets differentially regulated in AP and OA-AP. **(A)** Venn diagram shows common differentially regulated gene targets in AP and OA-AP. **(B)** KEGG pathway enrichment analysis shows the top 20 terms based on enrich factor arrangement. **(C)** GO analysis shows the top 20 terms based on enrich factor arrangement.

Supplementary Figure S5 CQCQD improved the apoptosis of pancreatic acinar cells. **(A)** Representative Western-blot images for Bax and Bcl2 in the pancreatic tissues. **(B)** Quantification of Western-blot images for Bax and Bcl2 for the pancreatic tissues. **(C)** Representative TUNEL statining for apoptosis in the pancreatic tissue. All data are presented as means ± SEM of 6 samples per group. Ctrl vs. OA-AP: **p* < 0.05; OA-AP vs. OA-AP + CQCQD: ^###^ *p* < 0.001.

Supplementary Figure S6 Interaction network of common differentially regulated gene targets in AP and OA-AP.
