# Supplementary material for: Transcriptomics and Network Pharmacology Reveal the Protective Effect of Chaiqin Chengqi Decoction on Obesity-Related Alcohol-Induced Acute Pancreatitis via Oxidative Stress and PI3K/Akt Signaling Pathway
Source: Front Pharmacol. 2022 Jun 8;13:896523. doi: 10.3389/fphar.2022.896523 (PMC9213732; doi:10.3389/fphar.2022.896523)
Supplement: Supplementary file 1 [file DataSheet1.pdf]

Figure S1

A

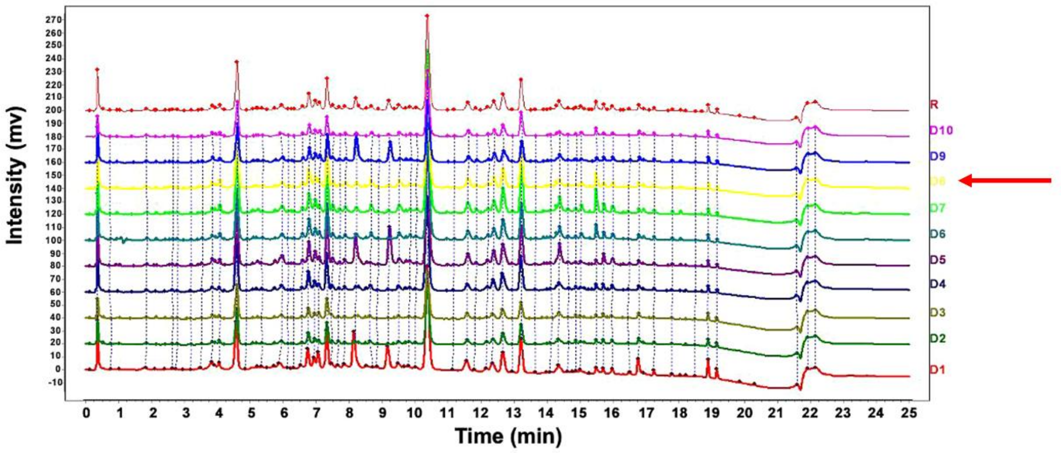

UHPLC fingerprint of CQCQD (Phytomedicine2021 PMID: 33740732)

B

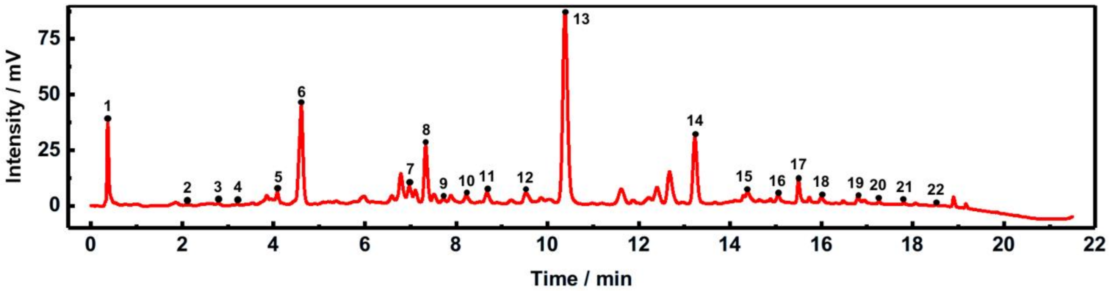

The representative total ion chromatograms (TIC) of CQCQD(D8)

Figure S2

Condition	Score	Indication
Edema	0	Absent
	1	Focally increased between lobules
	2	Diffusely increased between loblues
	3	Acini disrupted
	4	Acini separated
Inflammation (inflammatory cell infiltrate)	0	Absent
	1	In ducts (around ductal margins)
	2	In the parenchyma (in **<20%** of the lobules)
	3	In the parenchyma (in **20%-50%** of the lobules)
	4	In the parenchyma (in **>50%** of the lobules)
Necrosis (acinar necrosis)	0	Absent
	1	Periductal necrosis (**<5%**)
	2	Focal parenchymal necrosis (**5%-20%**)
	3	Diffuse parenchymal necrosis (**20%-50%**)
	4	Diffuse parenchymal necrosis (**>50%**)

Figure S3

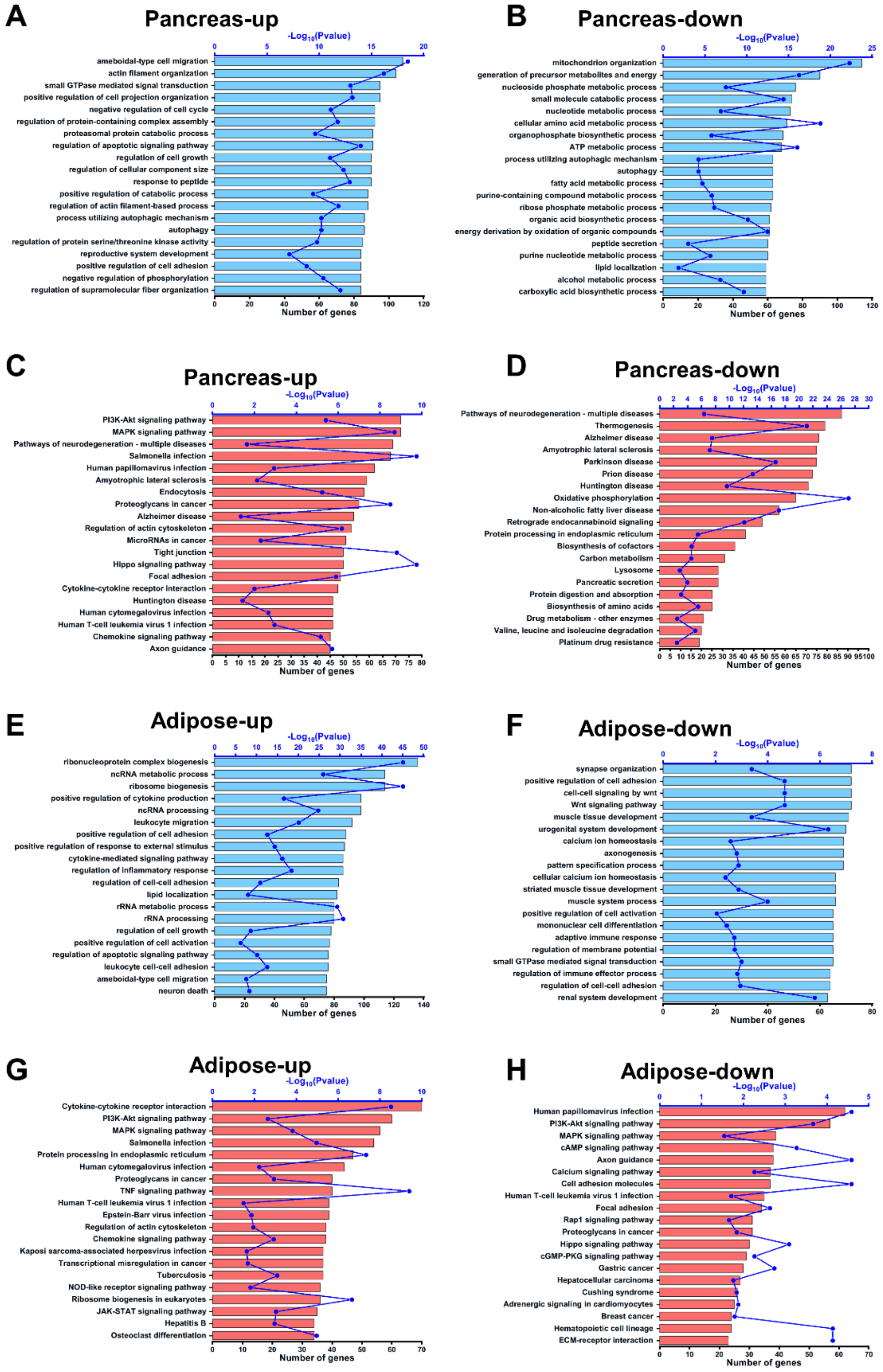
A
Pancreas-up
-Log10(Pvalue)
ameboidal-type cell migration
actin filament organization
small GTPase mediated signal transduction
positive regulation of cell projection organization
negative regulation of cell cycle
regulation of protein-containing complex assembly
proteasomal protein catabolic process
regulation of apoptotic signaling pathway
regulation of cell growth
regulation of cellular component size
response to peptide
positive regulation of catabolic process
regulation of actin filament-based process
process utilizing autophagic mechanism
autophagy
regulation of protein serine/threonine kinase activity
reproductive system development
positive regulation of cell adhesion
negative regulation of phosphorylation
regulation of supramolecular fiber organization
Number of genes
B
Pancreas-down
-Log10(Pvalue)
mitochondrion organization
generation of precursor metabolites and energy
nucleoside phosphate metabolic process
small molecule catabolic process
nucleotide metabolic process
cellular amino acid metabolic process
organophosphate biosynthetic process
ATP metabolic process
process utilizing autophagic mechanism
autophagy
fatty acid metabolic process
purine-containing compound metabolic process
ribose phosphate metabolic process
organic acid biosynthetic process
energy derivation by oxidation of organic compounds
peptide secretion
purine nucleotide metabolic process
lipid localization
alcohol metabolic process
carboxylic acid biosynthetic process
Number of genes
C
Pancreas-up
-Log10(Pvalue)
PI3K-Akt signaling pathway
MAPK signaling pathway
Pathways of neurodegeneration - multiple diseases
Salmonella infection
Human papillomavirus infection
Amyotrophic lateral sclerosis
Endocytosis
Proteoglycans in cancer
Alzheimer disease
Regulation of actin cytoskeleton
MicroRNAs in cancer
Tight junction
Hippo signaling pathway
Focal adhesion
Cytokine-cytokine receptor interaction
Huntington disease
Human cytomegalovirus infection
Human T-cell leukemia virus 1 infection
Chemokine signaling pathway
Axon guidance
Number of genes
D
Pancreas-down
-Log10(Pvalue)
Pathways of neurodegeneration - multiple diseases
Thermogenesis
Alzheimer disease
Amyotrophic lateral sclerosis
Parkinson disease
Prion disease
Huntington disease
Oxidative phosphorylation
Non-alcoholic fatty liver disease
Retrograde endocannabinoid signaling
Protein processing in endoplasmic reticulum
Biosynthesis of cofactors
Carbon metabolism
Lysosome
Pancreatic secretion
Protein digestion and absorption
Biosynthesis of amino acids
Drug metabolism - other enzymes
Valine, leucine and isoleucine degradation
Platinum drug resistance
Number of genes
E
Adipose-up
-Log10(Pvalue)
ribonucleoprotein complex biogenesis
ncRNA metabolic process
ribosome biogenesis
positive regulation of cytokine production
ncRNA processing
leukocyte migration
positive regulation of cell adhesion
positive regulation of response to external stimulus
cytokine-mediated signaling pathway
regulation of inflammatory response
regulation of cell-cell adhesion
lipid localization
rRNA metabolic process
rRNA processing
regulation of cell growth
positive regulation of cell activation
regulation of apoptotic signaling pathway
leukocyte cell-cell adhesion
ameboidal-type cell migration
neuron death
Number of genes
F
Adipose-down
-Log10(Pvalue)
synapse organization
positive regulation of cell adhesion
cell-cell signaling by wnt
Wnt signaling pathway
muscle tissue development
urogenital system development
calcium ion homeostasis
axonogenesis
pattern specification process
cellular calcium ion homeostasis
striated muscle tissue development
muscle system process
positive regulation of cell activation
mononuclear cell differentiation
adaptive immune response
regulation of membrane potential
small GTPase mediated signal transduction
regulation of immune effector process
regulation of cell-cell adhesion
renal system development
Number of genes
G
Adipose-up
-Log10(Pvalue)
Cytokine-cytokine receptor interaction
PI3K-Akt signaling pathway
MAPK signaling pathway
Salmonella infection
Protein processing in endoplasmic reticulum
Human cytomegalovirus infection
Proteoglycans in cancer
TNF signaling pathway
Human T-cell leukemia virus 1 infection
Epstein-Barr virus infection
Regulation of actin cytoskeleton
Chemokine signaling pathway
Kaposi sarcoma-associated herpesvirus infection
Transcriptional misregulation in cancer
Tuberculosis
NOD-like receptor signaling pathway
Ribosome biogenesis in eukaryotes
JAK-STAT signaling pathway
Hepatitis B
Osteoclast differentiation
Number of genes
H
Adipose-down
-Log10(Pvalue)
Human papillomavirus infection
PI3K-Akt signaling pathway
MAPK signaling pathway
cAMP signaling pathway
Axon guidance
Calcium signaling pathway
Cell adhesion molecules
Human T-cell leukemia virus 1 infection
Focal adhesion
Rap1 signaling pathway
Proteoglycans in cancer
Hippo signaling pathway
cGMP-PKG signaling pathway
Gastric cancer
Hepatocellular carcinoma
Cushing syndrome
Adrenergic signaling in cardiomyocytes
Breast cancer
Hematopoietic cell lineage
ECM-receptor interaction
Number of genes

Figure S4

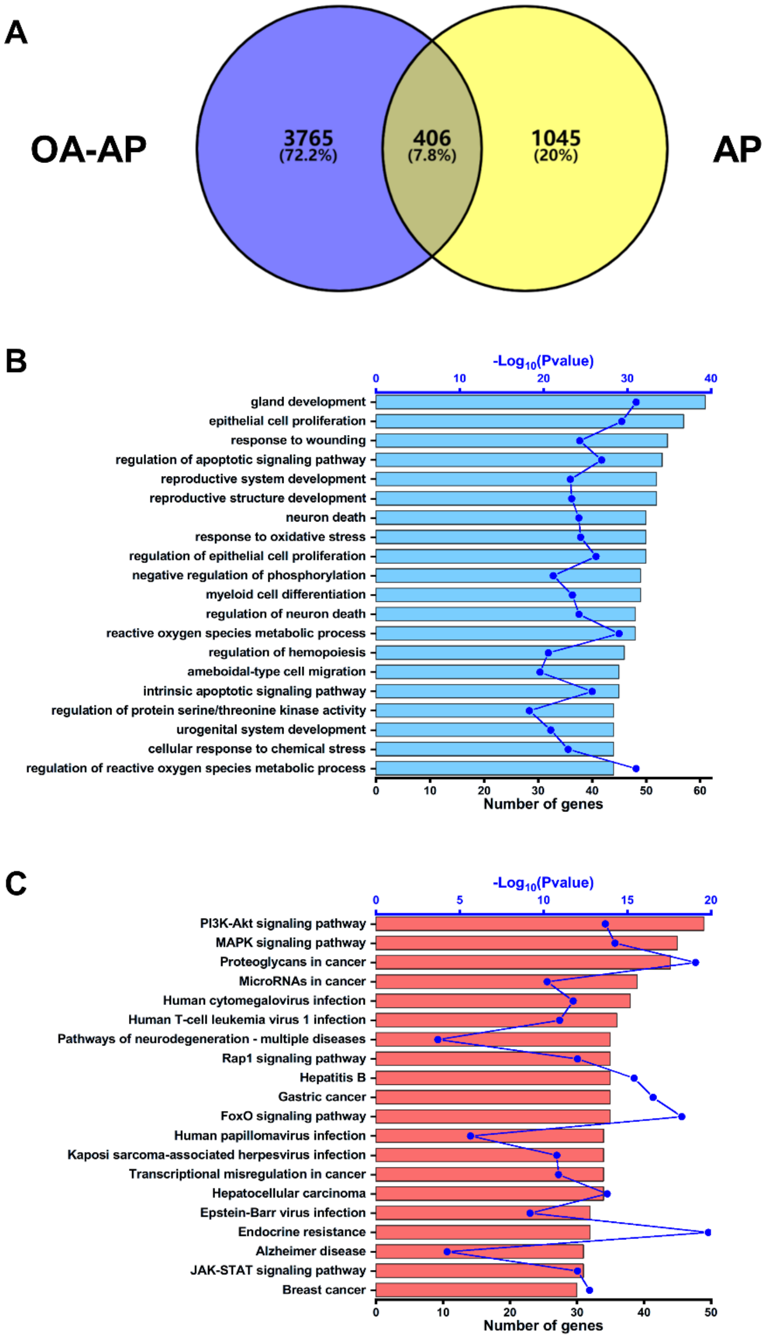
A
OA-AP
3765
(72.2%)
406
(7.8%)
1045
(20%)
AP
B
-Log10(Pvalue)
gland development
epithelial cell proliferation
response to wounding
regulation of apoptotic signaling pathway
reproductive system development
reproductive structure development
neuron death
response to oxidative stress
regulation of epithelial cell proliferation
negative regulation of phosphorylation
myeloid cell differentiation
regulation of neuron death
reactive oxygen species metabolic process
regulation of hemopoiesis
ameboidal-type cell migration
intrinsic apoptotic signaling pathway
regulation of protein serine/threonine kinase activity
urogenital system development
cellular response to chemical stress
regulation of reactive oxygen species metabolic process
Number of genes
C
-Log10(Pvalue)
PI3K-Akt signaling pathway
MAPK signaling pathway
Proteoglycans in cancer
MicroRNAs in cancer
Human cytomegalovirus infection
Human T-cell leukemia virus 1 infection
Pathways of neurodegeneration - multiple diseases
Rap1 signaling pathway
Hepatitis B
Gastric cancer
FoxO signaling pathway
Human papillomavirus infection
Kaposi sarcoma-associated herpesvirus infection
Transcriptional misregulation in cancer
Hepatocellular carcinoma
Epstein-Barr virus infection
Endocrine resistance
Alzheimer disease
JAK-STAT signaling pathway
Breast cancer
Number of genes

Figure S5

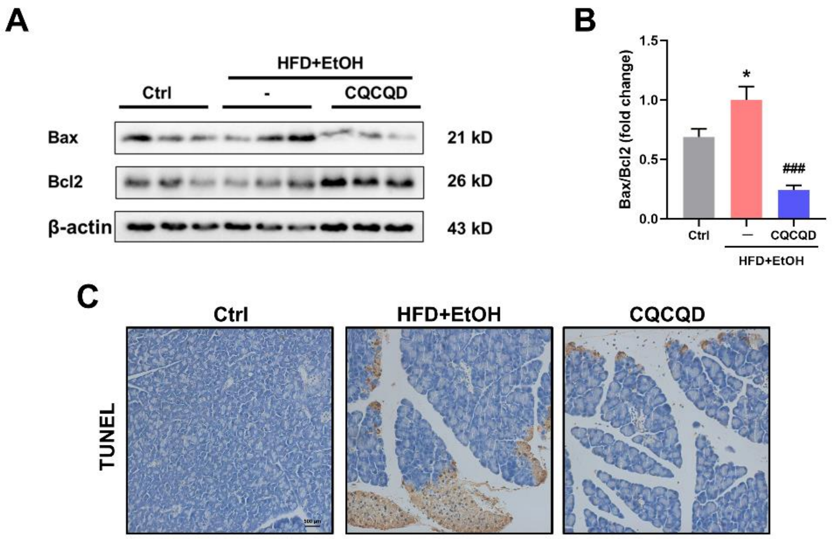
A
HFD+EtOH
Ctrl
-
CQCQD
Bax
Bcl2
β-actin
21 kD
26 kD
43 kD
B
Bax/Bcl2 (fold change)
1.5
1.0
0.5
0.0
*
###
Ctrl
CQCQD
HFD+EtOH
C
Ctrl
HFD+EtOH
CQCQD
TUNEL

Figure S6

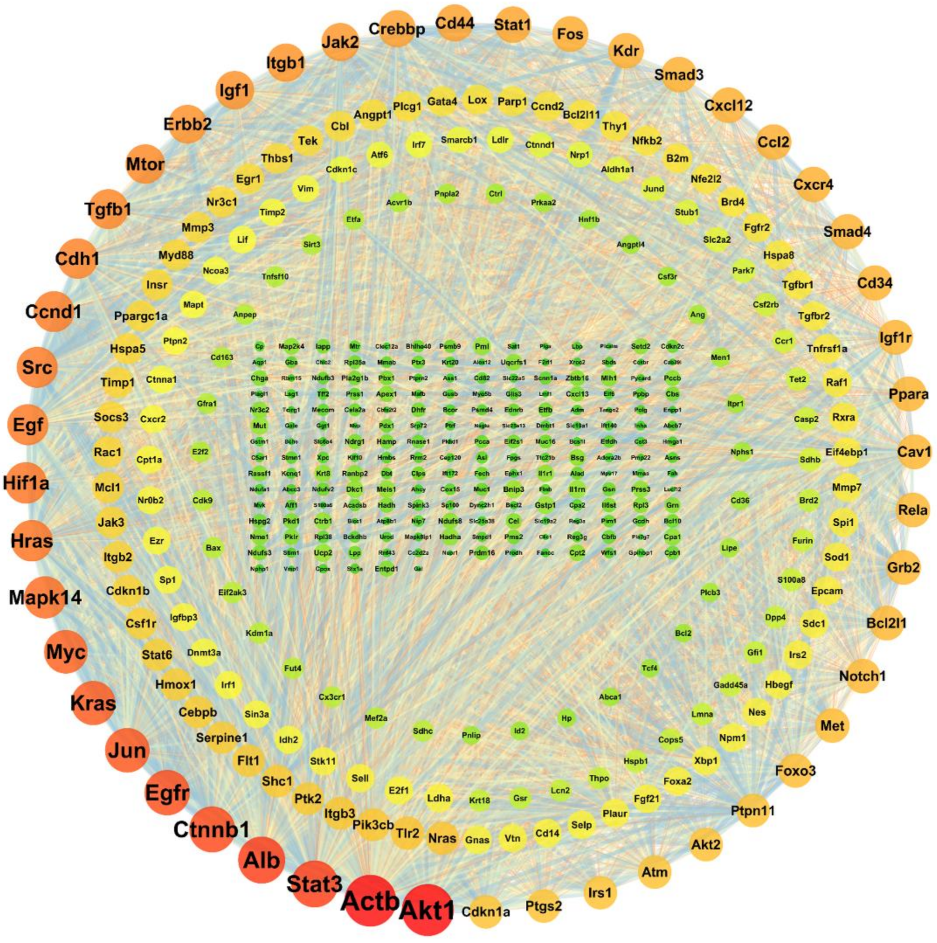

Jak2
Crebbp
Cd44
Stat1
Fos
Kdr
Smad3
Cxcl12
Ccl2
Cxcr4
Smad4
Cd34
Igf1r
Ppara
Cav1
Rela
Grb2
Bcl2l1
Notch1
Met
Foxo3
Ptpn11
Akt2
Atm
Irs1
Ptgs2
Cdkn1a
Akt1
Actb
Stat3
Alb
Ctnnb1
Egfr
Jun
Kras
Myc
Mapk14
Hras
Hif1a
Egf
Src
Ccnd1
Cdh1
Tgfb1
Mtor
Erbb2
Igf1
Itgb1
Plcg1
Gata4
Lox
Parp1
Ccnd2
Bcl2l11
Thy1
Angpt1
Cbl
Tek
Thbs1
Egr1
Nr3c1
Mmp3
Myd88
Insr
Ppargc1a
Hspa5
Timp1
Socs3
Rac1
Mcl1
Jak3
Itgb2
Cdkn1b
Csf1r
Stat6
Hmox1
Cebpb
Serpine1
Flt1
Shc1
Ptk2
Itgb3
Pik3cb
Tlr2
Nras
Gnas
Vtn
Cd14
Selp
Plaur
Fgf21
Foxa2
Xbp1
Npm1
Nes
Hbegf
Irs2
Sdc1
Epcam
Sod1
Spi1
Mmp7
Eif4ebp1
Rxra
Raf1
Tnfrsf1a
Tgfbr2
Tgfbr1
Hspa8
Fgfr2
Brd4
Nfe2l2
B2m
Nfkb2
